# ChatGPT in education: global reactions to AI innovations

**DOI:** 10.1038/s41598-023-42227-6

**Published:** 2023-09-15

**Authors:** Tim Fütterer, Christian Fischer, Anastasiia Alekseeva, Xiaobin Chen, Tamara Tate, Mark Warschauer, Peter Gerjets

**Affiliations:** 1 Hector Research Institute of Education Sciences and Psychology, University of Tübingen, Europastraße 6, 72072 Tübingen, Germany; 2 University of California, Irvine, USA; 3 Leibniz-Institut für Wissensmedien, Tübingen, Germany

**Keywords:** Psychology, Human behaviour

## Abstract

The release and rapid diffusion of ChatGPT have caught the attention of educators worldwide. Some educators are enthusiastic about its potential to support learning. Others are concerned about how it might circumvent learning opportunities or contribute to misinformation. To better understand reactions about ChatGPT concerning education, we analyzed Twitter data (16,830,997 tweets from 5,541,457 users). Based on topic modeling and sentiment analysis, we provide an overview of global perceptions and reactions to ChatGPT regarding education. ChatGPT triggered a massive response on Twitter, with education being the most tweeted content topic. Topics ranged from specific (e.g., cheating) to broad (e.g., opportunities), which were discussed with mixed sentiment. We traced that authority decisions may influence public opinions. We discussed that the average reaction on Twitter (e.g., using ChatGPT to cheat in exams) differs from discussions in which education and teaching–learning researchers are likely to be more interested (e.g., ChatGPT as an intelligent learning partner). This study provides insights into people's reactions when new groundbreaking technology is released and implications for scientific and policy communication in rapidly changing circumstances.

## Introduction

Artificial intelligence (AI) has the potential to transform the field of education, and its applications are becoming increasingly prevalent^1^. The massive diffusion and adoption of ChatGPT following its November 30, 2022 release suggest that AI can rapidly change how we learn and communicate. The release of ChatGPT generated a great deal of excitement and trepidation as to its possible effects on education^2^. The announcement from Microsoft to make programs like ChatGPT available for all users through its office programs^3^ hints at the broad impact of how people may soon leverage AI in their written communication. As generative AI tools such as ChatGPT become more integrated into education^4^, educators must address crucial questions about the future of teaching and learning. Students will need to understand how AI works, its affordances and challenges, and how they can harness its power without reproducing the biases inherent in its training data. Teachers will have to walk alongside, learning as they go and reinforcing preexisting habits such as corroboration and interrogation of sources, critical thinking, and ethical use of sources.

Notably, the spread and speed of innovation often depend on its usage by early adopters and their perception of the new technology^5^. Therefore, in this paper, we explore the global reception of ChatGPT in the first two months after its release. We aim to understand how the global educational community viewed the potential impact of ChatGPT on education and human learning. This may include topics from the potential to personalize learning to the ethical implications of relying on AI for information and communication. Specifically, we leverage social media data on Twitter to analyze the worldwide reception of ChatGPT, seeking insight into (a) the most prevalent topics discussed regarding ChatGPT in education and (b) how users discussed these topics over this initial implementation period.

## Theoretical background

### What is ChatGPT?

ChatGPT (https://openai.com/blog/chatgpt) is the latest release of the Generative Pre-trained Transformer (GPT) family of language models released by OpenAI (https://openai.com) on November 30, 2022. A language model is a statistical model that can predict the probability of a sequence of words. With this capability, a language model can generate natural language in a human style. Like all statistical models, a language model needs to be trained by many word sequences to calculate the probability of each sequence. The number of word sequences or the training corpus size used to train a model determines how much experience a model can gain about the language and, more importantly, the knowledge incorporated in the language. ChatGPT is a large language model trained with data from the Internet and many scanned books. Brown et al.^6^ reported using a corpus of 499 billion words to train the GPT-3 model, which was the base model for ChatGPT at its first release. ChatGPT now draws on GPT-4, a much larger and more powerful model. The GPT models are transformer models, allowing downstream fine-tuning for improved performance on more specific tasks, such as conversations or document classification. The conversation fine-tuning that ChatGPT obtained on top of GPT-3 aims at reducing untruthful, toxic, or unhelpful output that uncontrolled large language models may produce^7^. The fine-tuning approach used in ChatGPT was called Reinforcement Learning with Human Feedback (RLHF). This method fine-tuned the original model with data annotated by human raters as more or less appropriate responses. Details of the fine-tuning process are reported in Ouyang et al.^7^ (see also https://openai.com/blog/chatgpt).

### Opportunities and risks of ChatGPT for education

ChatGPT and other large language models can potentially have a large effect on teaching and learning in practice. This may include, for instance, the potential to facilitate more personalized and adaptive learning^8,9^ and organize assessment and evaluation processes more efficiently^4,8,10^. Also, Kasneci et al.^2^ emphasize the potential to compensate for educational disadvantages. Using speech-to-text technologies or automated generation of written texts, visual impairments or partial impairments such as dyslexia can be less limiting in learning, contributing to inclusive education. Zhai^21^ looked at the *Next Generation Science Standards* and tested how teachers could use ChatGPT to overcome key instructional challenges, such as providing feedback and learning guidance or recommending learning materials to students. For educational purposes, the specific potential of ChatGPT lies in its interactive component that allows for the execution of effective learning mechanisms. For instance, feedback is a core feature of learner support that is effective in learning^11,12^. ChatGPT can be understood as a learning partner or teaching assistant that gives feedback if good prompts are given by the learners^13–15^. Organizations such as the Khan Academy are quickly trying to exploit the power of ChatGPT as a learning partner by integrating the tool with prompts already built into their platform (see www.khanacademy.org/khan-labs).

Such education opportunities contrast with AI’s limitations and associated risks. One urgent limitation of ChatGPT is that no source of truth was included during the Reinforcement Learning training of ChatGPT (https://openai.com/blog/chatgpt). Thus, the risk that ChatGPT will produce texts with plausible-sounding but incorrect information is high^8–10,13–18^. Educators and learners need professional knowledge and critical reflection skills to assess the responses generated adequately^19^ (see also 21st-century skills^20^). ChatGPT as a learning partner, may not promote critical thinking per se^21^. However, use guided by educators can provide opportunities for critical thinking (e.g., as students learn to refine prompts based on their understanding of the context or genre). The question of how ChatGPT (and its successors) opportunities for education can and should be best exploited and how its risks can be best avoided is an important and interdisciplinary research issue that will unfold over the next few years.

### Human reactions to groundbreaking technological innovations

New technologies like ChatGPT can only achieve their potential if they are used in pedagogically sound ways. Active use is likely if users have a positive attitude toward the technology. Numerous theories postulate the importance of rational processes for accepting and adapting technological innovations. For instance, the Technology Acceptance Model (TAM;^22^, which is based on the Theory of Reasoned Action^23,24^ and the Theory of Planned Behavior^25,26^, is a frequently used model to describe the actual use of technology^27^. Thus, these theories and models provide a framework for conceptualizing human behavior in the context of technological innovations. However, besides cognitive theories describing rational processes and non-rational behaviors like biases or misconceptions, humans' emotions, and feelings are also important factors determining why people adapt to new technologies^5,28^. For instance, social science research has shown that emotions are a crucial factor for decision-making processes (e.g., functioning as helpful guides or as bias factors; Lerner et al.^29^,) or consumer behavior^30^ (for more studies, see Valor et al.^28^,).

Regarding ChatGPT, a first ad hoc study on user sentiment towards the tool is available based on Twitter data for December 5–7, 2022^31^. The authors identified the *impact of ChatGPT on education* as one primary topic users talked about with mixed sentiment (52% positive, 32% negative, 16% neutral). Statements made by individual education stakeholders shortly after ChatGPT was released reflect this mixed sentiment. It is interesting, however, that the positive tweets somewhat outweigh the negative tweets overall because equally, just the opposite (i.e., predominantly negative tweets) would have been plausible when thinking about the fixed routines in many educational institutions that are potentially challenged using ChatGPT (e.g., performance assessment). However, these findings are only based on data from the first two days after the release of ChatGPT. Whereas some educational stakeholders express apprehension about ChatGPT, other educational stakeholders are optimistic about the introduction of ChatGPT^32^. For instance, individual statements by students and scientists illustrate that, on the one hand, there is a fear that students will outsource their writing and thinking when using ChatGPT or that proven routines such as commonly used homework or assessment methods (e.g., essays) can now no longer be used by educators. On the other hand, the opportunity for a transformation of educational processes is seen, for instance, by redesigning written assessments in such a way that less important skills, such as superficial text features such as grammar or spelling, are pushed into the background allowing critical and logical thinking skills to come to the fore.

### Twitter data as a measure to gain insights into human reactions

Twitter is a microblogging platform where registered users can post brief messages called tweets (up to 280 Unicode characters for non-paying users; tweets). Tweets can be linked to specific persons (through links [@]) or specific topics (via hashtags [#]). Users can follow other users' tweets. Twitter provides access to real-time data that can capture the public perceptions on innovations like ChatGPT (another example CRISPR^33^), significant events (e.g., Arab spring, U.S. elections, COVID-19:^34,35^), or reforms^36^. Twitter data has affordances as a research tool as it provides scalable and unbiased data that is also fine-grained from a temporal perspective^37^. Therefore, Twitter data is potentially better suited for understanding innovations on a larger scale than more time-consuming traditional research methods like surveys or interviews.

### Aims and research questions

ChatGPT can potentially transform educational processes worldwide, but whether and how it does so may depend on how educators take it up. In this study, we aim to gain insights into an unvarnished and immediate global human reaction to the release of ChatGPT that goes beyond statements made by individual stakeholders in education. Our study may help estimate human reactions to technology innovations that can also be relevant to the education sector in the future (e.g., incorporating measures for acceptance of new educational technologies such as information on benefits or guidelines on how to use this technology directly when they are introduced). In addition, we examine which education-related topics were discussed by users and which topics tend to be disregarded but should not be ignored in a critical discussion of ChatGPT in educational contexts. We focused on the following three research questions (RQs) in the first two months after the ChatGPT release (November 30, 2022):

*(RQ1) What was the global reception on Twitter about ChatGPT?*

*(RQ2) What topics related to ChatGPT emerged in the education topical cluster during this initial period on Twitter?*

*(RQ3) How were the most prevalent educational topics related to ChatGPT discussed on Twitter?*

## Methods

### Data collection and preparation

Using the Twitter API for Academic Research, we collected 16,830,997 tweets posted from November 30, 2022, to January 31, 2023. We chose this rollout period to get an initial reaction from the public before many had spent much time thinking about or using ChatGPT. The data collection procedure was as follows: first, we queried the Tweets that mentioned *ChatGPT*. Second, we identified and collected all conversations with either a *sparking* tweet (i.e., tweets that start a discussion) or a reply mentioning ChatGPT. A conversation is defined as the sparking tweet and all other tweets directly replying to the sparking tweet. Notably, a conversation needs to include at least two different users. This led to 127,749 unique conversations. Notably, we found no mentions of ChatGPT on Twitter before November 30, 2022.

We anonymized the data to protect users’ privacy by replacing Twitter-assigned tweet, author, and conversation identifiers with random numeric identifiers. Email addresses and phone numbers were substituted with placeholders. Similarly, we replaced usernames with anonymous user identifiers. In addition, we removed tweets that were most likely generated by computer programs (i.e., bots) using unsupervised text-based and heuristics approaches. First, we removed bots by removing those 257,858 accounts that posted more than ten tweets during the observation period, or contained the word *bot* in their screen name, or their screen name ended with *app* or included *app* followed by a non-letter symbol (*self-declared bots*). We set the number of tweets threshold based on the assumption that bots are prone to tweet, on average, significantly more than humans^38^. We also removed accounts from the dataset that posted more than 1000 tweets. Overall, 283 bots and their 80,389 tweets were deleted based on the first rule. Second, we deleted repetitive tweets about unrelated topics (e.g., spam-like product advertisements or cryptocurrency). The groups of tweets were found by clustering the document (tweet) embeddings. This text-based approach is preferred over the available tools for bot detection, such as *Botometer*^39^, because of the large dataset size and heterogeneous nature of the data. In addition, modern bots are prone to behave in groups rather than individually. The text-based approach can capture coordinated behavior^40^. This led to a final sample size of 16,743,036 tweets, 5,537,942 users, and 125,151 conversations.

### Analytical methods

#### Topic modeling

We applied a topic modeling procedure to gain insight into topics users discussed after ChatGPT was released (RQ1 and RQ2). We selected only tweets in English, deleted empty tweets and duplicates, and removed user mentions starting with “@”, hashtags, and links in all tweets. We deleted the term *ChatGPT* and its derivatives to improve the model performance, as this term appeared in all tweets (due to the inclusion criteria of our data collection:^41^). Next, we used a BERTopic algorithm^41^ to retrieve clusters of similar tweets from the dataset. The algorithm allows using document embeddings generated by state-of-the-art language models. It outperforms conventional topic modeling techniques such as Latent Dirichlet Allocation (LDA) and Non-Negative Matrix Factorization (NMF), as it accounts for semantic relationships among words and provides better topic representations^41,42^.

Moreover, BERTopic was used successfully in recent studies on Twitter^43,44^. We used a Python implementation of the BERTopic algorithm (https://github.com/MaartenGr/BERTopic) with the minimum size of a cluster set at 500 and 3 different language models: BERTweet (https://huggingface.co/docs/transformers/model_doc/bertweet), twitter-roberta-base-sep2022 (https://huggingface.co/cardiffnlp/twitter-roberta-base-mar2022), and all-MiniLM-L6-v2 (https://huggingface.co/sentence-transformers/all-MiniLM-L6-v2). We examined the performance of each embedding model on our dataset by reviewing 20 tweets from each topic. The last embedding model showed a better performance regarding topic diversity and coherence. We ran the model on all *non-conversation tweets* written in English, not including retweets (i.e., 520,566 sparking and other non-conversation tweets). We did not include retweets as they decelerate clustering significantly while adding little value to the output of topic modeling. Then, we extrapolated the results on retweets (526,780 full-text retweets) using supervised classification and manually grouped some of these topics into larger topical clusters.

#### Sentiment analysis

We performed sentiment analysis to gain insight into how users discussed ChatGPT after it was released (RQ1 and RQ3). For this, we used all tweets in English, including conversations. The preprocessing procedure was identical to the one applied at the topic modeling step. Next, we used the rule-based model VADER to perform the sentiment analysis^45^, as VADER showed a high accuracy on Twitter data^46^ and outperformed other sentiment software like LIWC when using Twitter data on education^47^. In addition, we excluded outliers (i.e., tweets identified within the topic modeling procedure that are far from other topics) to achieve a more accurate estimate of sentiment for education-related tweets.

We make our data preparation and analysis syntaxes freely available at the following link: https://github.com/twitter-tuebingen/ChatGPT_project.

### Ethical approval

An ethics committee approved the study and the collection of the data. It confirmed that the procedures aligned with ethical research standards with human subjects (date of approval: 09-02-2023, file number: AZ.: A2.5.4-275_bi).

## Results

### The global reception of ChatGPT (RQ1)

**Figure 1:**
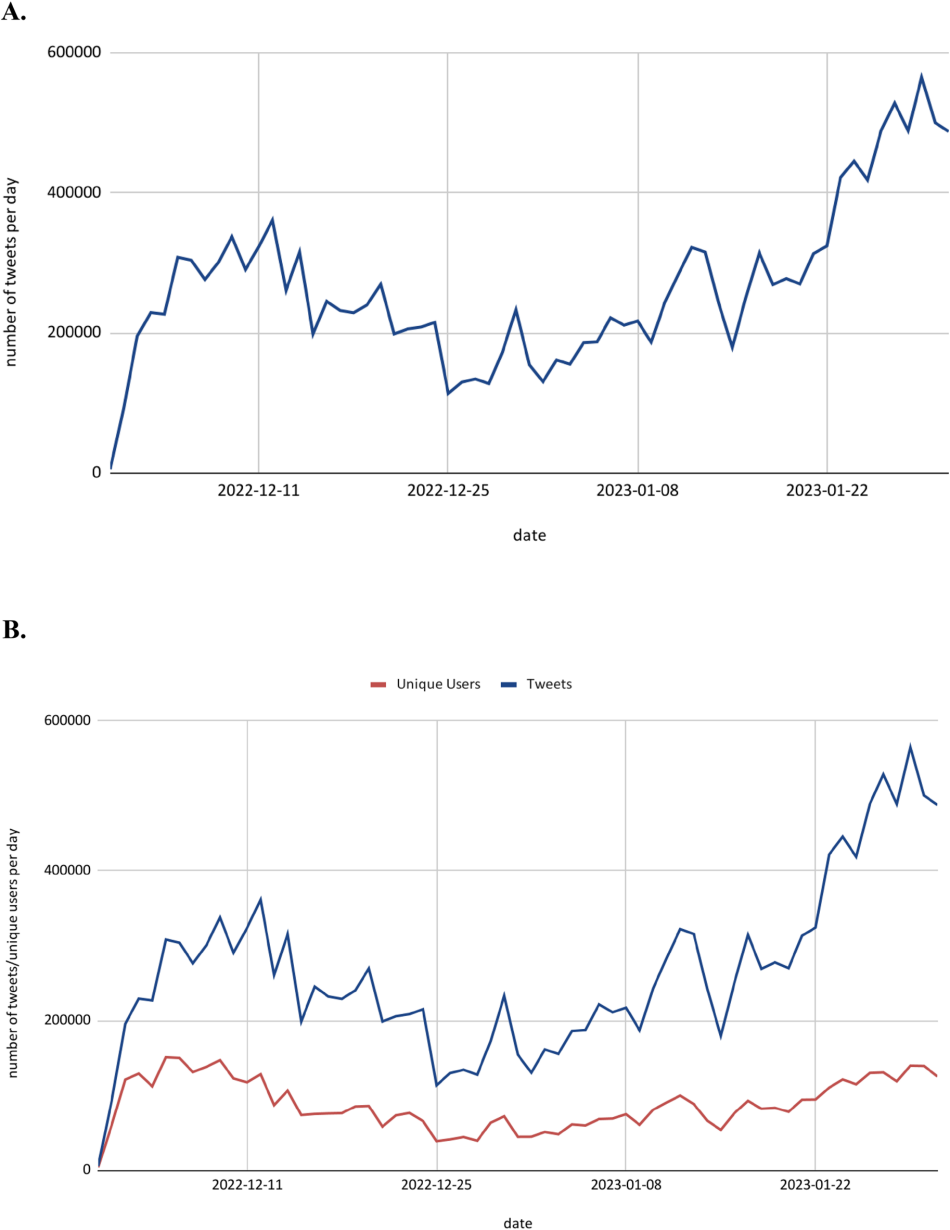
(**A**) Number of tweets per day dealing with ChatGPT. (**B**) Unique users and number of tweets per day dealing with ChatGPT. We used all 16,743,036 tweets.

To gain insights into the global reception on Twitter about ChatGPT, we first looked at all 16,743,036 tweets (without identified bots and spam) of the first two months after the release of ChatGPT that include the term *ChatGPT* and tweets in related conversations (for descriptive statistics regarding the number of likes, retweets, replies, and quotes see Table 1A,B). We found that the number of tweets per day increased on average from 0 tweets before November 30, 2022, to over 550,000 tweets per day at the end of January (Fig. 1A,B). This number is impressive compared to the number of tweets related to other prominent hashtags in the past. For instance, in their analyses of the social media response (i.e., Twitter) to the Black Lives Matter debate, Ince et al.^48^ found that the Hashtag #BlackLivesMatter (including different spellings) was mentioned 660,000 times from the beginning of 2014 to November 2014. A more current comparison is the number of tweets regarding vaccine manufacturers AstraZeneca/Oxford, Pfizer/BioNTech, and Moderna during the COVID-19 pandemic. From December 1, 2020, to March 31, 2021, Marcec and Likic^49^ retrieved 701,891 tweets in English.

**Table 1:** Descriptive statistics of likes, retweets, replies, and quotes.

	*M*	Median	*SD*		min	25%	50%	75%	Max	
(A) All tweets^a^										
Likes	4.568	0	236.468		0	0	0	1	176,848	
Retweets	229.981	0	1688.009		0	0	0	0	35,579	
Replies	0.391	0	13.761		0	0	0	0	17,185	
Quotes	0.036	0	3.449		0	0	0	0	4612	
(B) Sparking tweets^b^										
Likes	76.837	4	972.979		0	1	4	16	159,790	
Retweets	13.026	0	186.667		0	0	0	2	35,568	
Replies	6.556	1	100.024		1	1	1	3	17,185	
Quotes	1.708	0	27.571		0	0	0	0	4612	

^a^These statistics refer to all *N* = 16,743,036 tweets from November 30, 2022, to January 31, 2023.

^b^These statistics refer to all *N* = 125,151 tweets from November 30, 2022, to January 31, 2023.

**Figure 2:**
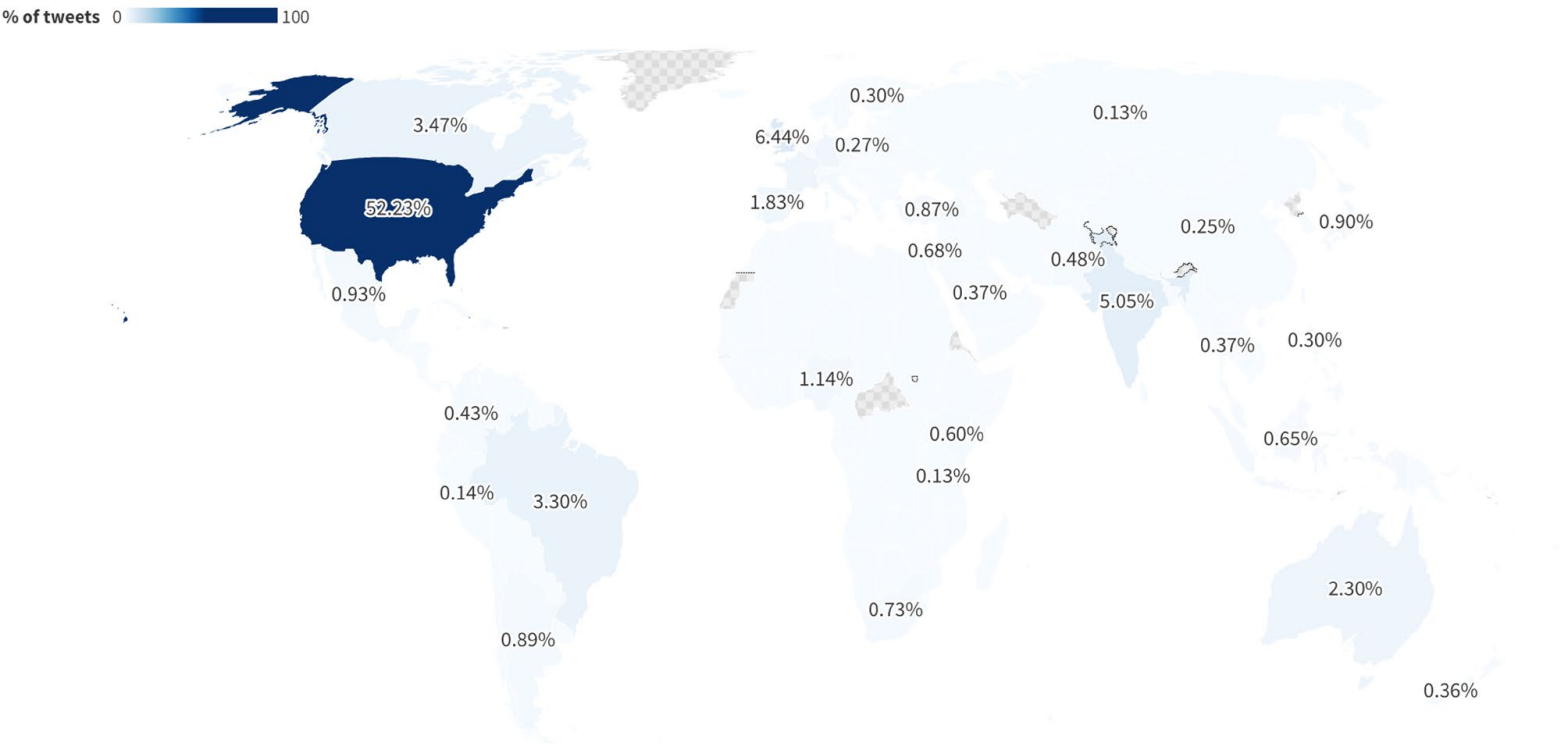
Global distribution of tweets. The visualization is based on 1% (i.e., 160,260) tweets with known locations out of 16,743,036 tweets. For an interactive version of this figure, see https://public.flourish.studio/visualisation/13026492/.

Most of the tweets related to ChatGPT (72.7%) were in English (see Fig. A in the appendix), and 52.2% came from the U.S. (Fig. 2). Next, we looked at the sentiment ratio of daily tweets (Fig. B, see also Fig. C in the appendix). Almost all tweets were positive in the very first days after the release of ChatGPT. However, the level (i.e., the proportion of tweets classified as *positive*, *neutral,* or *negative*) then flattened, remaining relatively stable with small overall fluctuations throughout the first two months. Whereas tweets classified as positive dominated all analyzed days within the first two months after the release of ChatGPT, the daily number of positive, neutral, and negative tweets converged to a 40–30–30 distribution over time. This distribution may suggest that users increasingly discussed ChatGPT more deliberately and reflectively, considering not only its impressive capacity but also the challenges that it poses. It is unsurprising for early technology adopters to be more positive than those subsequently investigating the technology. The sentiment change may partially reflect a more diverse universe of tweet authors.

### Topics related to ChatGPT in education (RQ2)

To gain insights into the topics discussed related to ChatGPT, we first ranked all 128 topics users discussed in our sample (i.e., gained from topic modeling) by the number of associated tweets. Having identified 128 topics indicates that the discussion about ChatGPT on Twitter touches upon many topics. Second, we manually grouped these topics into 39 larger topical clusters based on semantic and cosine similarities. *Education* was the third most prevalent topical cluster (measured by the number of tweets; Table 2) after discussions of general tweets about AI (the most prevalent topical cluster) and tweets that contain *examples of questions and prompts* (the second most prevalent topical cluster).

**Table 2:** Topics overall.

Topic	Volume	% Total	Average length of conversation
AI in general	114,830	10.96	6.31
Question examples, prompt engineering	104,529	9.98	5.71
Education	86,934	8.30	5.19
OpenAI and its Investors and products (Microsoft, Musk)	80,077	7.65	5.58
Access and price	77,795	7.43	9.55
LLM technology	70,433	6.72	6.22
Impact on search engines	53,557	5.1	5.85
Impact on art (poems and lyrics, movies, books)	47,672	4.55	4.69
Digital content generation(podcasts,youtube scripts,quizzes)	41,671	3.98	5.50
Programming	35,648	3.40	4.84
Business routine	34,425	3.29	5.70
Entertainment	26,144	2.50	4.82
Cybersecurity (writing malware)	24,869	2.37	11.17
ChatGPT on social media	22,707	2.17	8.17
Finance	22,076	2.11	6.35
Healthcare	19,199	1.83	7.17
Emotional reaction (scary, insane)	16,375	1.56	4.20
Politics	13,931	1.33	5.48
Legal issues	11,377	1.09	5.89
Making money with ChatGPT	10,981	1.05	7.49
Social events on ChatGPT, discussion on media	9178	0.88	3.80
Recipes	8015	0.77	5.38
Calculator, math	7843	0.75	5.09
Climate change	7807	0.75	58.84
Job loss	7802	0.74	7.36
Criticism in terms of ethics	7769	0.74	5.13
Text to audio/voice	7143	0.68	5.08
Spam	6219	0.59	4.33
Sport	4828	0.46	4.33
Robots	4028	0.38	3.63
Religion, sermons	3821	0.36	4.71
Q&A platforms	3776	0.36	6.06
Christmas	3406	0.33	3.18
Translation	2787	0.27	4.11
Gender	2337	0.22	5.53
Real estate	2242	0.21	5.36
ChatGPT's competitors	2170	0.21	4.64
Quantum computing	1853	0.18	4.47
Outliers (small volume topics)	39,092	3.73	8.65

1,047,346 tweets used (i.e., English non-conversation tweets). Volume indicates the absolute number of tweets.

*LLM* large language models.

An overview of the ten most prevalent topics in education discussed on Twitter is given in Table 3. The most prevalent topic in the education topical cluster consisted of statements regarding the opportunities, limitations, and consequences of using ChatGPT in educational processes in general (Table 3). These statements comprised 22% of all conversations (see T1 in Table 3). For instance, the functions of ChatGPT for educational processes were discussed (e.g., getting feedback), and measures for a successful implementation of ChatGPT in educational processes were discussed (e.g., prerequisites for educators and learners, such as awareness of opportunities and boundaries of ChatGPT and an awareness of ethical aspects). The second most prevalent topic in education consisted of statements related to efficiency and cheating when students use ChatGPT to, for instance, do homework like essays. These statements comprised 18% of all conversations (see T2 in Table 3).

**Table 3:** The most important topics in education.

Topic (T)	Volume	%	Anchor tweets (synthetic)
T1. Opportunities, limitations, and consequences of the use of ChatGPT	27,368	22.49	a. #chatgpt is an incredible learning tool. It's like working with a senior developer who has limitless patience and whom you can ask all your questions. What an amazing time to live! b. Being creative and proactive educators, our priority is to support our students in becoming ethical and responsible users of these tools so that they can graduate as technologically literate citizens.
T2. Efficiency and cheating when students use ChatGPT to write (e.g., homework like essays)	21,526	17.69	a. My bro used ChatGPT to write an essay for college and just banged it out in 10 s using ChatGPT… b. The rise of AI in cheating. ChatGPT is able to write compelling, sophisticated essays.
T3. Opportunities and limitations of ChatGPT in academia (e.g., writing research papers)	19,654	16.15	a. Abstracts written by ChatGPT can fool scientists! b. I asked ChatGPT about the topic of my PhD. It generated reasonably looking explanations and references until I fact-checked the references…
T4. Ban ChatGPT in educational organizations	12,084	9.93	a. The NYC public schools are banning ChatGPT on school networks and devices b. The NYC Education Department made a short-sighted decision. It is the same as if we had banned calculators when they first came out.
T5. Capability of ChatGPT to pass exams	9107	7.49	a. ChatGPT took my exam on Microeconomics and flunked. 12/100 for the exam b. ChatGPT passing the Wharton MBA proves that an MBA is the most pointless activity you can engage in
T6. Strategies to use ChatGPT for writing	7090	5.82	a. Tip: Before writing an essay, ask ChatGPT to generate one on the same topic to find out the typical thing to say so that you can avoid it b. My child, after asking ChatGPT to write an essay, said: “It’s producing what I’d say, but mine is better because it provides more depth”. Educators should be relaxed about kids using AI. Treat it more like giving ideas
T7. Other AI tools for education and developments in the future	6422	5.28	a. ChatGPT is the tip of the iceberg. 10 AI tools that will change education forever! b. The technology behind #ChatGPT is creating new startups and services every day. These 7 AI-powered websites are revolutionizing education!
T8. The capability of ChatGPT to replicate students' papers	5220	4.29	a. It is impossible for ChatGPT to imitate the charm of a college paper written by an undergraduate student who did not attend classes or read lecture materials. The student's pure determination and desperation allow them to create a unique piece of writing that artificial intelligence could never replicate! b. AI can't create. It can only replicate what has already been created.
T9. Costs of education	4413	3.63	a. Thanks to AI. You can learn anything you want, for free! No need to spend thousands of dollars on a course. b. If both teachers and students use ChatGPT to complete/judge challenges, there is no need to go to universities. Schools are more than Q/A; they are places for findings and discussions.
T10. How educators could integrate ChatGPT in teaching	2457	2.02	a. NEW!!! How Can Educators Leverage ChatGPT to Save Time? b. Have you tried ChatGPT for your classroom? #teachers #chatgpt
Outliers	6325	5.20	

121,666 tweets used (i.e., English non-conversation tweets plus English-related conversation tweets). Volume indicates the absolute number of conversations. The volume was used as an indicator of the importance of the topics. Anchor tweets are synthetic tweets that represent typical content of the topics exemplarily.

Similarly, the role of ChatGPT in academia was discussed (the third most prevalent topic in education, covering 16% of all conversations; see T3 in Table 3). For instance, on the one hand, opportunities of using ChatGPT (e.g., support in the standardized process of writing a research paper) and the limitations (e.g., no references or made-up references, potential danger for academia if ChatGPT is not used reflectively) were addressed. The fourth prevalent topic was banning ChatGPT in educational organizations (covering 10% of all conversations; see T4 in Table 3). Although there were discussions worldwide about regulations such as bans in schools and universities, the news that ChatGPT was banned from New York City public schools' devices and networks, in particular, dominated discussions on Twitter. In addition to these four dominant topics, which together covered 66% of the total education-related conversations, the topics T5 (*capability of ChatGPT to pass exams*), T6 (*strategies for using use ChatGPT for writing*), T7 (*other AI tools for education and developments in the future*), T8 (*the capability of ChatGPT to replicate students' papers*), and T9 (*costs of education*) each covered between 7.5% and 3.6% of all education-related conversations. The topic of how educators could integrate ChatGPT into teaching–learning processes (e.g., teachers in their lessons) was of minor importance (covering only 2% of all conversations; see T10 in Table 3).

**Figure 3:**
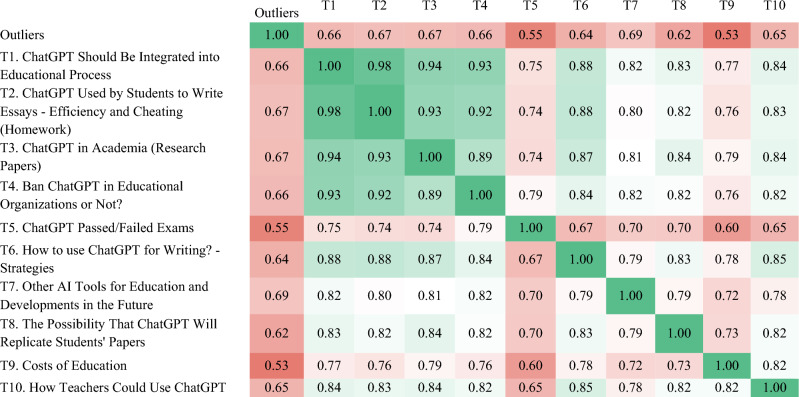
Pairwise cosine similarity between topic embeddings.

The pairwise cosine similarity between topic embeddings (Fig. 3) illustrates that the ten educational topics are closely related. Closely linked are the topics T1 (i.e., opportunities, limitations, and consequences of the use of ChatGPT), T2 (i.e., efficiency and cheating when students use ChatGPT to write [e.g., homework like essays]), T3 (i.e., opportunities and limitations of ChatGPT in academia [e.g., writing research papers]) and T4 (i.e., ban ChatGPT in educational organizations). This means that the words chosen within the topics have significant commonalities. The close connection may also indicate that these four topics may have been discussed concurrently. For instance, the opportunities, limitations, and consequences of using ChatGPT were probably often discussed with multiple perspectives in mind. Considering, at the same time, how students will use ChatGPT to write essays, what challenges students and researchers face in writing papers, and what consequences should be drawn for regulations regarding the use of ChatGPT by organizations such as schools and universities. In contrast, the topics of how *ChatGPT can pass current exams* in various disciplines and *educational costs* had fewer commonalities with the other topics.

### Sentiments of most prevalent educational topics (RQ3)

**Figure 4:**
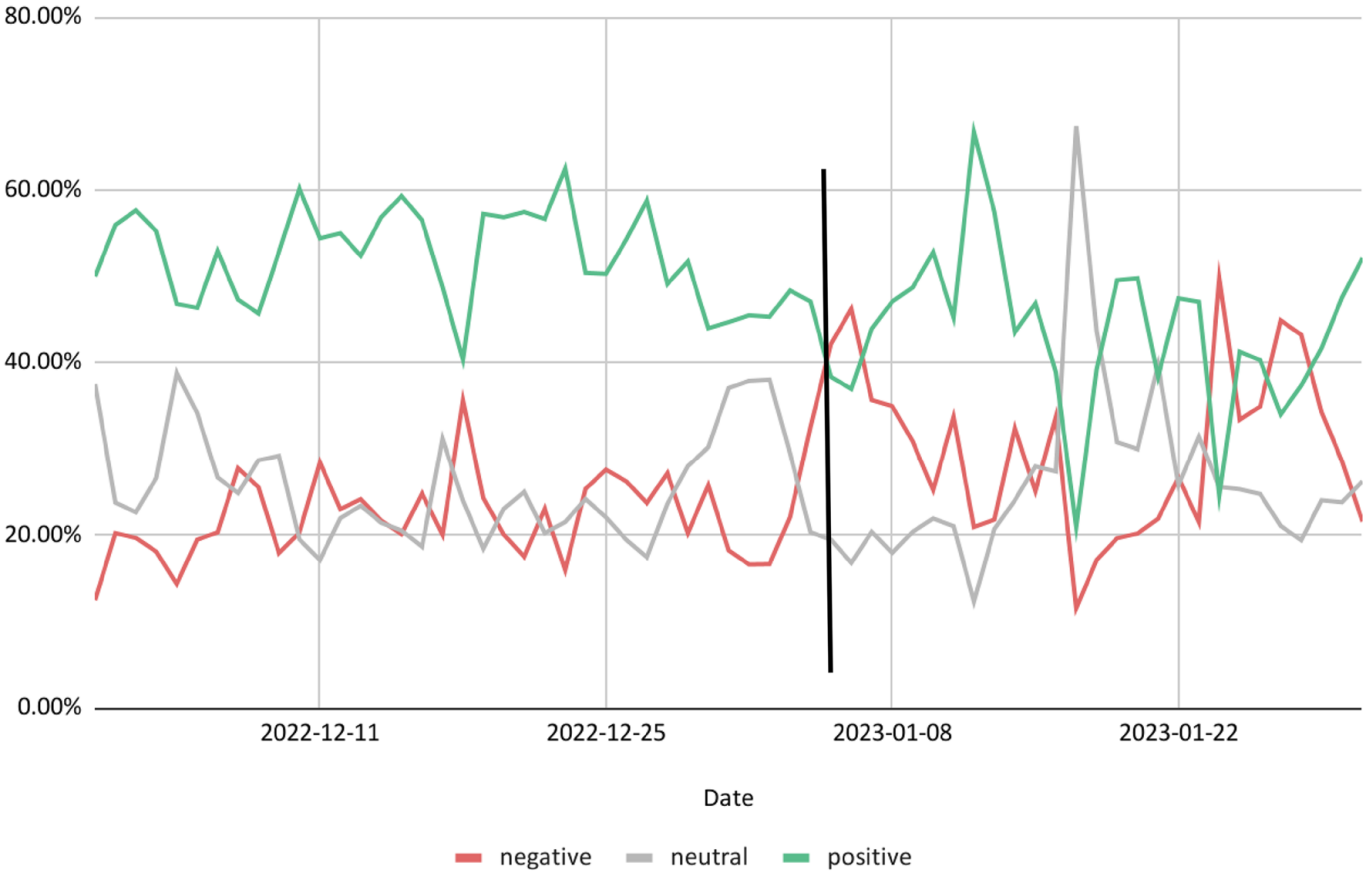
Sentiment in education per day. The vertical line is plotted on January 5, 2023, which was the day when the proportion of negative tweets was greater than the proportion of positive tweets for the first time.

To gain insights into how the most prevalent education topics related to ChatGPT were discussed, we examined the sentiments of all 86,934 tweets related to education (Table 2) and the related 34,732 conversation tweets (i.e., 121,666 tweets in total; Fig. 4). On average, the number of tweets with positive sentiment outweighed tweets with neutral and negative sentiment throughout the first two months after ChatGPT's release. Descriptively, positive tweets decreased, and negative and neutral tweets increased over the two months. Before January 5 (see the vertical line in Fig. 4), the largest share of tweets was positive every day after the release of ChatGPT. Only from January 5 onwards the shares of all three sentiments started to alternate so that the share of negative or neutral tweets was the highest on some days.

**Figure 5:**
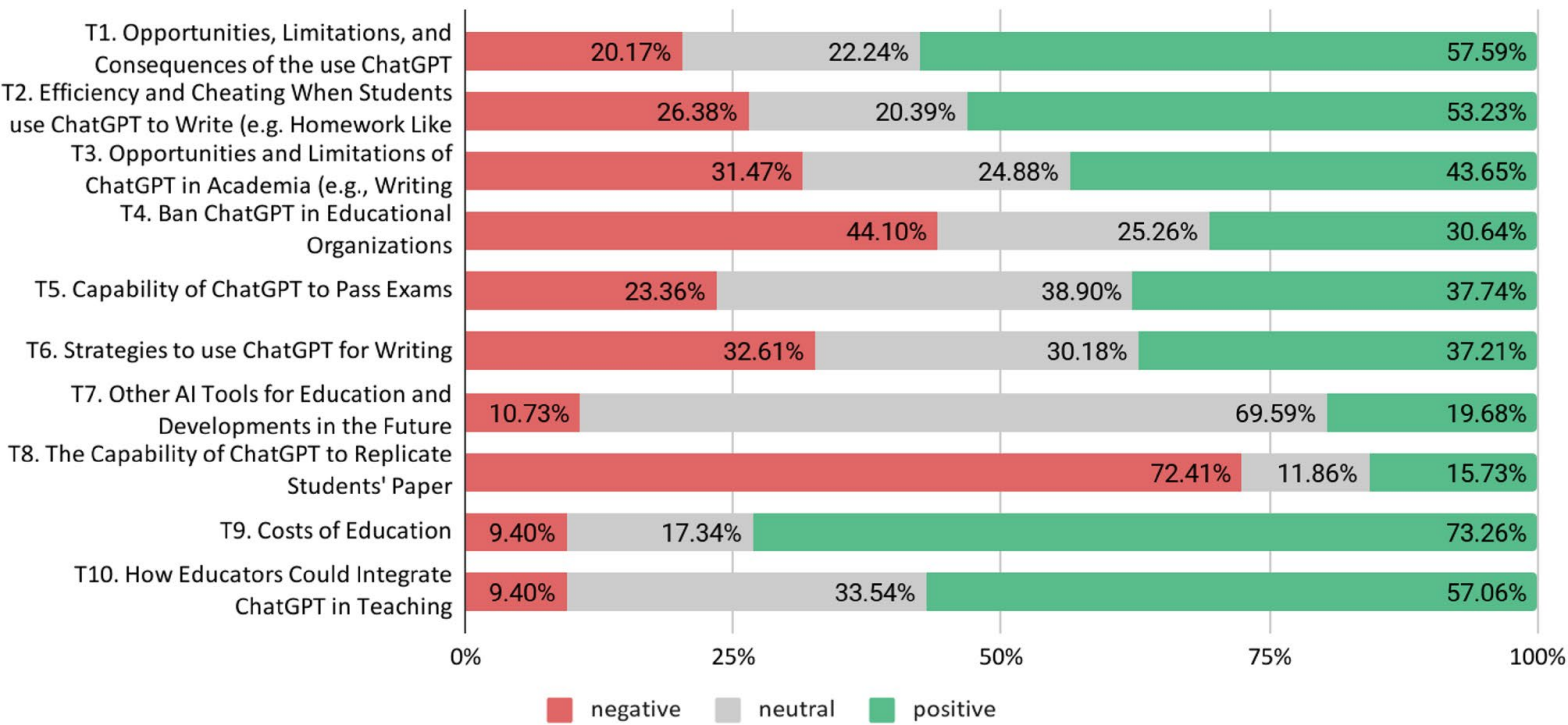
Sentiment in education by topic.

Sentiments on each of the ten identified topics are shown in Fig. 5. The costs of education resulting from using AI tools such as ChatGPT were discussed with the most positive sentiment (73.3%). This positive sentiment was mainly because many people anticipated a reduction in the cost of education through AI technology such as ChatGPT. The topic that was discussed the second most positively is how educators could use ChatGPT (57.1% positive sentiment). The positive statements referred especially to the possibility of saving time for educators. For example, the potential of ChatGPT to be used to create assignments for worksheets or exams was highlighted. Additional topics were discussed with a diverse sentiment (e.g., banning ChatGPT in educational institutions [44.1% negative, 25.3% neutral, 30.6% positive sentiment] or how to use ChatGPT for writing [32.6% negative, 30.2% neutral, 37.2% positive sentiment]). Whether ChatGPT will ever replicate student papers was discussed most negatively (72.4% negative sentiment). These negative statements appear to reflect the view that ChatGPT is only capable of producing schematic essays and is not as original or creative as human writers, thus incapable of generating new ideas.

## Discussion

We aimed to gain insights into an unvarnished and immediate global reaction to the release of ChatGPT, focusing on the education-related topics users discussed and the sentiment of these discussions. Therefore, we analyzed 16,830,997 tweets posted in the first two months after the release of ChatGPT that included the word ChatGPT.

First, regarding the global reception on Twitter about ChatGPT (RQ1), we traced the rapid awareness of ChatGPT shortly after its release. Whereas before November 30, 2022, not a single tweet worldwide contained the word *ChatGPT,* the rise of ChatGPT after its release was almost unstoppable, with more than 100,000 tweets per day at the beginning of December 2022 and over half a million by the end of January 2023. This massive worldwide awareness is also confirmed, for instance, the hits on Google (707,000,000 hits when searching *ChatGPT* on March 24, 2023) and the high number of users (4 months after its launch, there are now an estimated 100 million active monthly users:^18,50^) indicate that ChatGPT is likely going to have a lasting impact on personal and professional lives.

Second, education was the top content topic for the ChatGPT discussion beyond generic topics (e.g., how to access ChatGPT). This is surprising because ChatGPT could drastically change professional practice in many professions where creative text production is central (e.g., journalism, book authoring, marketing, business reporting). Implications include that educational stakeholders (e.g., school/higher education administrators, teachers/instructors, educational policy-makers) must develop guidelines for its use in their contexts.

Third, zooming in on education-related topics (RQ2), we found that both specific topics (e.g., students' essay writing, cheating, the capability of ChatGPT to pass exams) and broader issues (e.g., opportunities, limitations, and consequences of the use of ChatGPT) were discussed. These topics may provide initial directions for educational stakeholders to develop ChatGPT usage guidelines.

Fourth, although the findings indicated that ChatGPT was generally discussed positively in the first 2 months after ChatGPT's release, the statements regarding education were more mixed. This aligns with previous research on perceptions of technological innovations, which showed that users face most innovations with varying expectations and emotions. Expectations and emotions are associated with attitudes ranging from absolute conviction of the new technology's usefulness and a positive attitude (“radical techno-optimists”) to complete rejection of the new technology (“techno-pessimists”)^51^. Especially after January 5, when it was announced that ChatGPT would be banned from New York City public schools’ devices and networks, the sentiment of the discussion began to be more mixed. The mixed sentiment is not necessarily bad; many potentially positive and negative effects of using ChatGPT in education must be carefully weighed. At the same time, it is important to consider how rapid policy decisions, taken as a precaution without an opportunity to weigh evidence, can influence public debate on topics. This aspect is important because technologies are only used and thus enfold their potential (e.g., for educational processes) if users recognize their benefits^22^. The critical topic of how educators could integrate ChatGPT into teaching–learning processes (e.g., teachers in their lessons) was addressed only in a few tweets. This is interesting as the didactically meaningful integration of AI tools such as ChatGPT into teaching–learning processes at schools and universities will certainly be a key question for education research, teacher education, and daily practice. Moreover, as strong policy decisions such as complete bans also inform public opinion, they might render it hard for scientific opinions on opportunities of new technologies such as ChatGPT to be heard. For instance, some of the most critical educational and scientific challenges, like inequalities, heterogeneity, and adaptivity that might be alleviated with AI tools, were not at the core of the public debate.

Finally, zooming into the sentiments of education-related topics (RQ3), we found that, for instance, the costs of education and how educators could use ChatGPT for teaching were discussed positively (e.g., the possibility of saving time). In contrast, whether ChatGPT can replicate student papers has been discussed negatively. The negative sentiment among tweets on the replication of papers from students by ChatGPT was fed, among others, by statements in which it was expressed that a technology like ChatGPT cannot replace humans in writing believable human-like text (e.g., with the charm of humans writing). However, research shows that people quickly think they can distinguish human text from AI text due to overconfidence when they cannot but are merely guessing (e.g.,^52,53^). As awareness (measured by Tweet volume) grew, the range of sentiments increased. This likely reflects a broader audience becoming aware of the technology beyond early adopters and the increased time to consider the potential positive and negative consequences. It also may reflect an increased opportunity to use the tool, resulting in both an awareness of its potential and an ability to learn firsthand about its weaknesses.

### Limitations and future research

The results of this study must be interpreted considering at least the following limitations. First, although we have thoroughly prepared the data for the analyses, problems arise due to bots. Bots are computer programs that perform automated tasks, such as automatically retweeting tweets with a specific hashtag. Bots are challenging to detect because their behavior constantly changes as technology advances^40^. We used heuristics and text-based approaches to identify and remove tweets posted by bots from our data but cannot guarantee that tweets from bots are still present in the data used. Second, due to our limitation of Tweets to those in English, our geographical focus is around 57% North America. Thus, our findings may not be generalizable to other regions. Third, insight into the sample used for this study is limited by the information available through the Twitter API. For instance, we could not accurately determine whether politicians, academics, entrepreneurs, or opportunists tweeted. However, we were able to provide some general insights into the sample by analyzing, for instance, the sample’s experience with Twitter (operationalized by Twitter sign-up date) or the users’ reach (operationalized by number of followers; see Figs. D and E and Table A in the appendix). Fourth, we analyzed only the first two months of conversations on Twitter after the release of ChatGPT. This means that subsequent discussions based on more experience with ChatGPT (educational stakeholders had more time to figure out strengths and weaknesses and use ChatGPT for, e.g., teaching–learning scenarios) or that included GPT4 were not considered. However, it was precisely the approach of this study to capture the unvarnished, rather unreflective reaction of humans to groundbreaking technological innovations. Moreover, this study goes beyond previous findings on human reactions to ChatGPT, some of which only focused on the first few days after the release of ChatGPT (e.g.^31^).

Our research approach was designed to address the overall international response of humans to the release of ChatGPT, which encourages numerous potential future research directions. First, it would be interesting better to understand the interplay between science and public opinion. For instance, it would be interesting to investigate whether it is possible to find any indications that Twitter users refer to science in their statements on ChatGPT. For instance, users may cite research papers in the tweets (e.g., compared to COVID-19 vaccinations, where public opinions on benefits and risks were driven by scientists' daily explanation of new preprint research findings). Moreover, it would be interesting to gain insights into what scientists initially said about the potentials and risks of GPT and especially ChatGPT, and if these opinions are reflected in public opinion. Second, research needs to address the pedagogical qualities of human-AI interaction, which did not play a role in the global response on Twitter in our data. While recent research aligns with this finding, for instance, examining institutional websites to see what the references to ChatGPT refer to^54^, research that examines how AI-powered programs like ChatGPT can be used didactically meaningful and learning-effective (i.e., for high-quality teaching and learning). This may also include studies about best practices for using ChatGPT in teaching–learning situations, such as teaching effectiveness (e.g., cognitive activation, feedback), cognitive load, offloading, and adaptivity. Third, future research can address specific scenarios of using GPT in the classroom. This could include studies examining how ChatGPT could lead to innovative forms of instruction, for instance, asking, "What can ChatGPT do that we would never have been able to do before (e.g., have it write five different essays on a topic and let it discuss with learners about which one is the best and why)?”. Also, whereas people on Twitter were discussing using ChatGPT to cheat, studies should examine the distinction between learning and performance (e.g., learning to write versus doing a homework writing assignment). With a performance mindset, one can always cheat (e.g., use a calculator for homework). However, the main issue is not passing an exam or presenting an essay. It is about conveying knowledge on how to pass the exam or write an essay. These examples illustrate how a global human response to technological innovation might differ from a more scientifically informed response. However, human responses can help scientists to identify these blind spots in discussions better to explore and communicate them in the future. Finally, this study only provides insight into the initial human reactions to ChatGPT (the first 2 months after the release of ChatGPT). Therefore, future work is encouraged to gain insight into the long-term effects of ChatGPT. This could include exploring subgroup effects for subjects such as art, music, literature, STEM, and history, as learning about literature (e.g., writing styles or foreign languages) might afford entirely different GPT scenarios than learning about STEM. Furthermore, we encourage researchers to conduct surveys and interview studies targeting various stakeholder groups to gain additional insights into the use of ChatGPT and their perceived challenges and affordances. Indeed, a steady stream of questionnaire-based studies of varying quality has emerged quickly in the first months of 2023, offering valuable insights into how certain user groups perceive and interact with ChatGPT. However, while providing meaningful and interesting findings, these studies are limited to certain user groups with specific local contexts and certain aspects of their user experiences—aspects identified and studied through their respective questionnaires. For example, typical studies of this category analyzed the perceptions and expectations of 50 senior Computer Engineering students in Abu Dhabi^55^, 2100 students from a Ghanaian university^56^, or 288 participants from a convenience sample study at an Indian university^57^. Compared to these questionnaire studies on narrow and focused user groups, our research approach is much broader and more globally oriented to address research questions that these smaller, targeted questionnaire studies cannot adequately address. Specifically, we sought a comprehensive, worldwide compilation of active user responses (not passive answers to predefined questions) to ChatGPT in its initial weeks of existence. This was a specific time window when user reactions might have been still spontaneous and uninfluenced by the repeated pro and con arguments about GPT that have since then permeated media coverage. Nevertheless, there are quite a few similarities regarding the positive and negative evaluations that Twitter uses expressed in the initial phase of ChatGPT and that different user groups later reported in questionnaire studies. Soon it will probably be interesting to analyze the trajectories of different user perceptions and expectations regarding generative AI systems such as ChatGPT over the years. However, studying these trajectories requires a sequence of snapshots at different points in time, as we provided in our study for the birth period of ChatGPT.

## Conclusion

To conclude, ChatGPT is perhaps unique in how it exploded into the conversation and is only comparable to digital tools such as the Internet and computers that have proven to be of similar transformative magnitude. The availability of social media, particularly Twitter, allowed many people to learn about and discuss generative AI quickly. ChatGPT was also particularly impressive in its capabilities, far more functional, accessible, and conversational than other publicly available large language models. This ability for a worldwide conversation in real-time about the exploration of a potentially transformative digital tool is illustrated by the over half a million daily tweets we see only two months after ChatGPT’s release. This rapid awareness in a space with an active academic user base allowed educators to participate in learning about and exploring the use of this new tool (and for many, over an academic break period when they perhaps had time and capacity more than usual). For these reasons, it is comprehensible that education is the third most frequent topic of Tweets during this period, following how to access the tool, general AI, and sample prompts (use cases). Twitter allows technologically savvy educators to discuss new tools, share best practices, and try on policy positions among their peers. Twitter may not be representative of typical teachers. However, those using social media are likely to be among early adopters and thought leaders on using educational technology in their schools, so their Tweets may be a bellwether of things to come. When investigating new tools such as ChatGPT, communicating with peers can open our eyes to their challenges and opportunities. Hopefully, these conversations will continue into more differentiated and educationally relevant discussions as we gain more experience with generative AI tools.

### Supplementary Information

Supplementary Information.

## Data Availability

The datasets generated and analyzed during the current study are not publicly available as we use Twitter data that cannot be completely anonymized without significant effort (i.e., individuals are identifiable through the tweets) but are available from the corresponding author on a reasonable request.
