# Supplementary material for: ChatGPT in education: global reactions to AI innovations
Source: Sci Rep. 2023 Sep 15;13:15310. doi: 10.1038/s41598-023-42227-6 (PMC10504368; doi:10.1038/s41598-023-42227-6)
Supplement: Supplementary file 1 — Supplementary Information. [file 41598_2023_42227_MOESM1_ESM.docx]

**Appendix**

**Figure A**

*Top 10 Languages of Tweets*


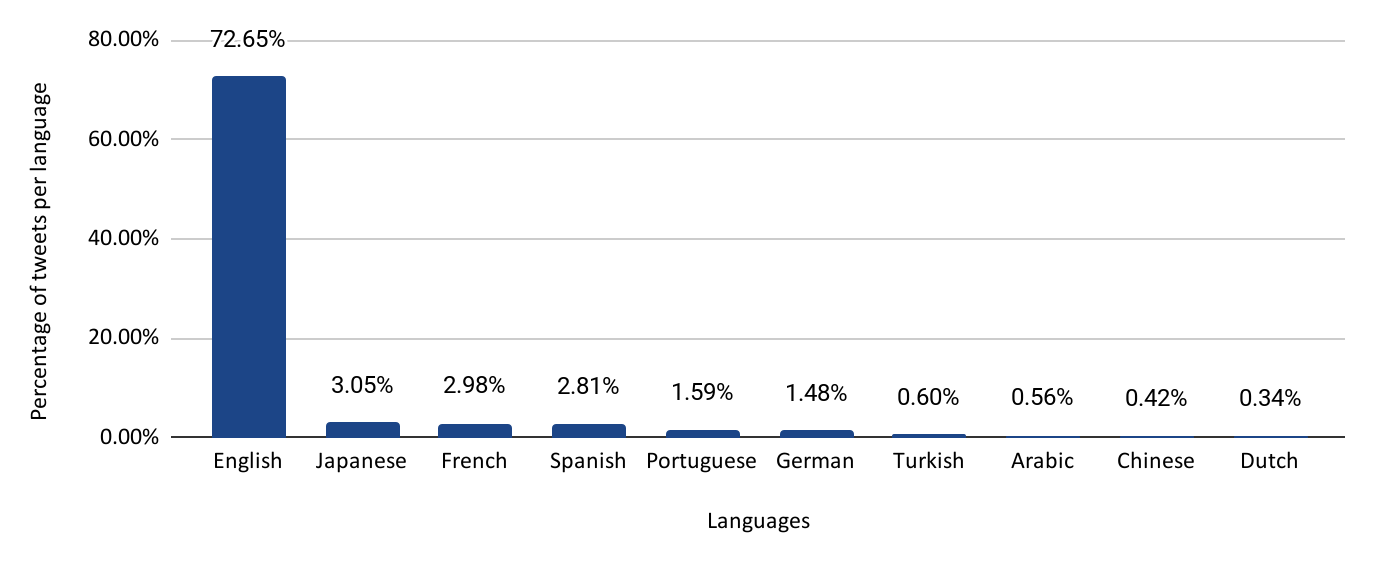


**Figure B**

*Sentiments of Tweets per day Dealing With ChatGPT*


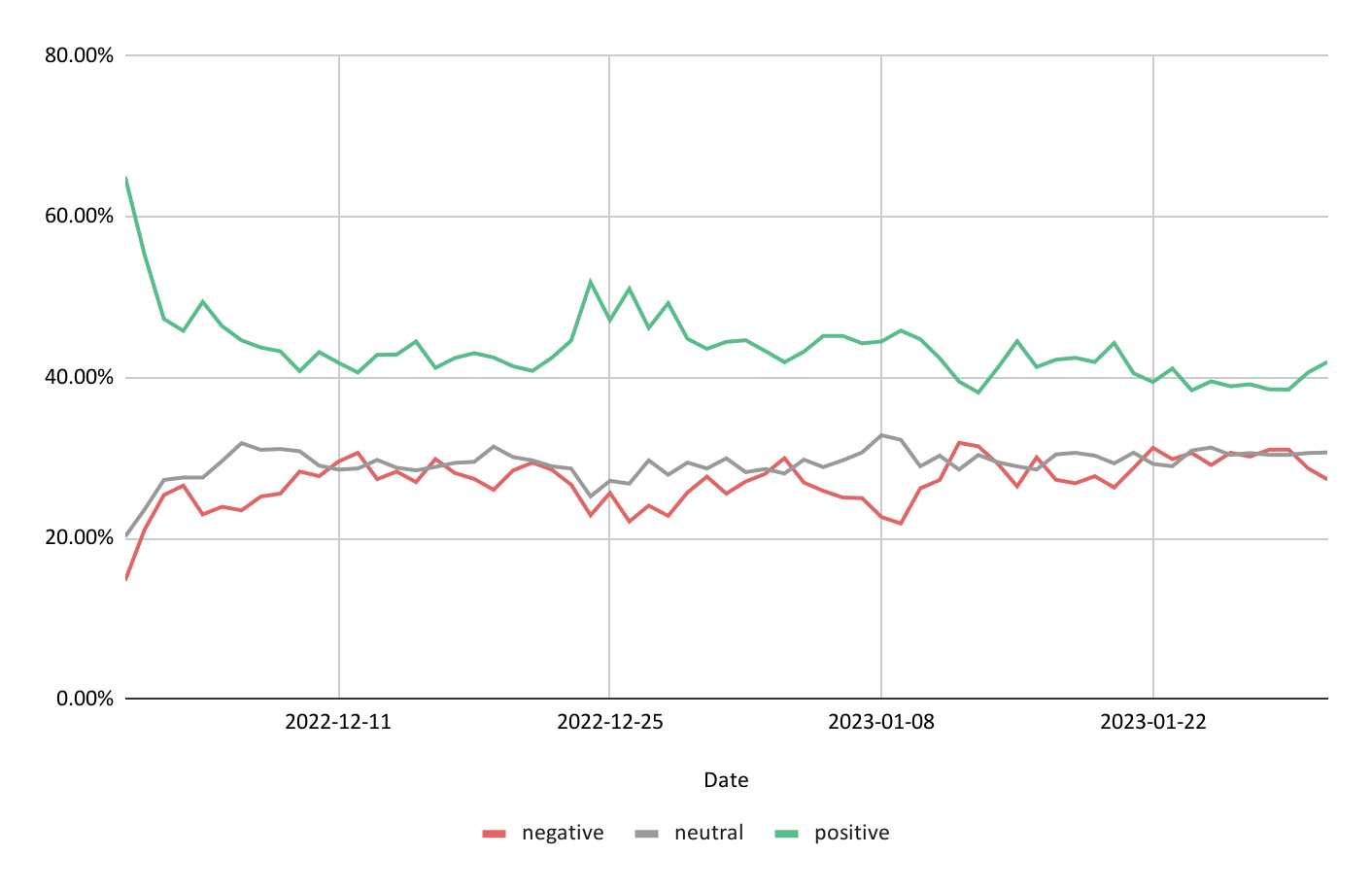


*Note.* We used 11,401,389 tweets (i.e., all tweets in English, including conversations).

**Figure C**

*Sentiments of Tweets per day Dealing With ChatGPT*


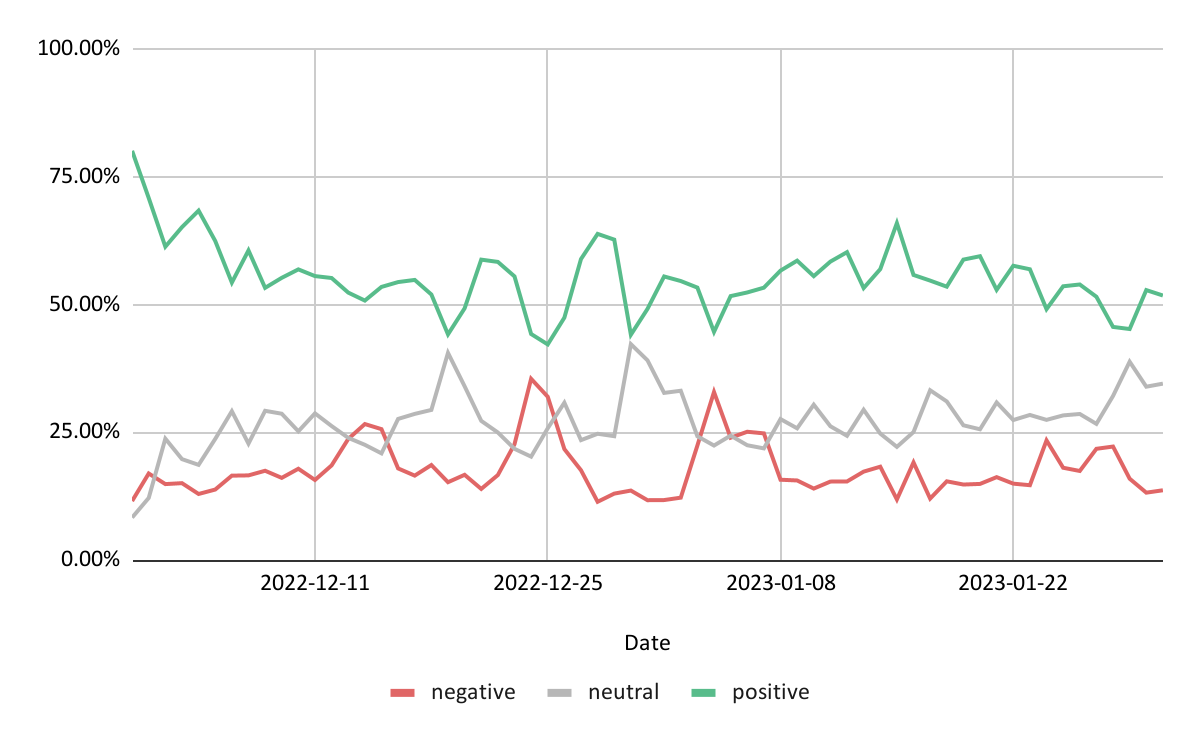


*Note.* 1,041,127 tweets used (i.e., all tweets in English, not including conversations).

**Figure D**

*Number of Accounts set up per Year*


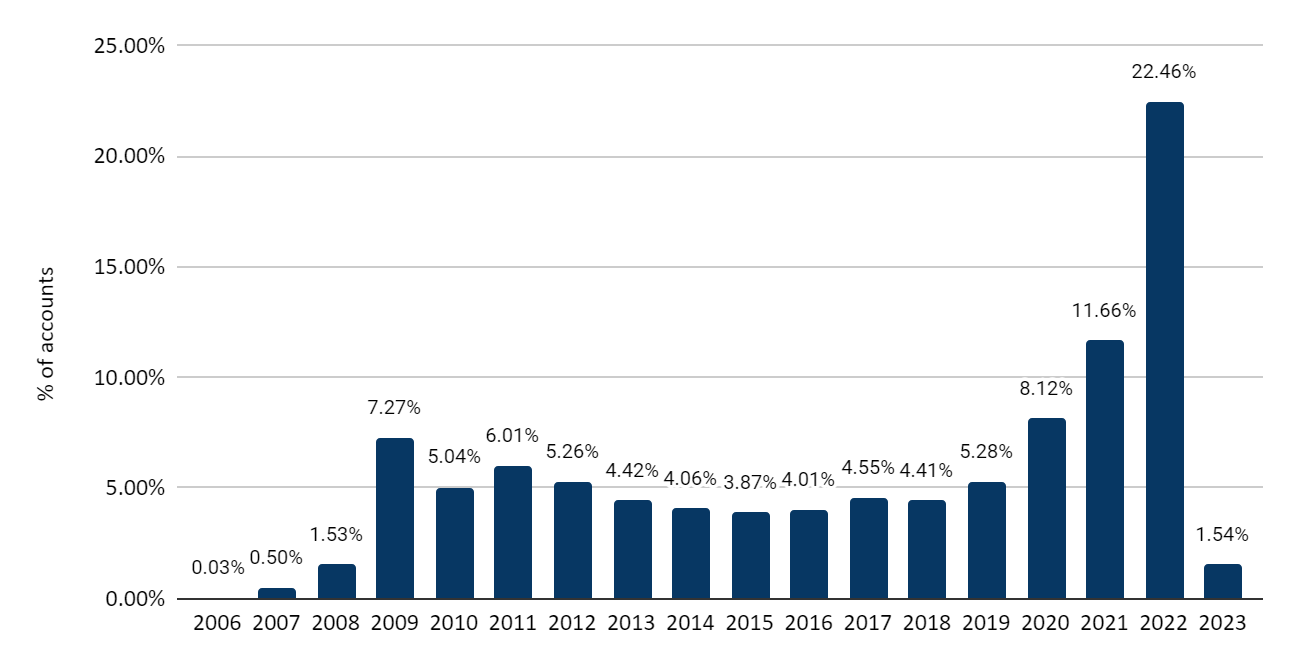


**Table A**

*Overview of the Number of Followers and Users Followed*

|  | Followers Count | Following Count |
| --- | --- | --- |
| *M* | 6,102.80 | 758.15 |
| *SD* | 260,089.77 | 4,333.44 |
| 25% | 15 | 81 |
| 50% | 84 | 255 |
| 75% | 404 | 717 |
| Max | 133,569,161 | 4,051,610 |

**Figure E**

*Wordcloud of top 100 Words in User Description*


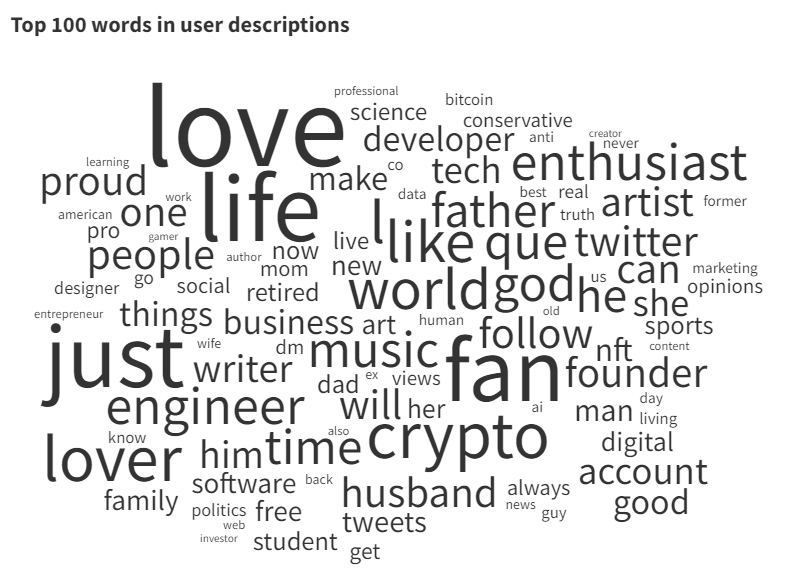


*Note.* The bigger the words are, the more often these words are used in the descriptions.
